# Recombinant purified buffalo leukemia inhibitory factor plays an inhibitory role in cell growth

**DOI:** 10.1371/journal.pone.0198523

**Published:** 2018-06-13

**Authors:** Syed Azmal Ali, Dhruba Malakar, Jai Kumar Kaushik, Ashok Kumar Mohanty, Sudarshan Kumar

**Affiliations:** Proteomics and Cell Biology Lab, Animal Biotechnology Center, National Dairy Research Institute, Karnal, Haryana, India; University of South Alabama Mitchell Cancer Institute, UNITED STATES

## Abstract

Leukemia Inhibitory Factor (LIF) is a polyfunctional cytokine, involved in numerous regulatory effects *in vivo* and *in vitro*, varying from cell proliferation to differentiation, and has therapeutic potential for treating various diseases. In the current study, a COS-1 cell line overexpressing recombinant Buffalo LIF (rBuLIF) was established. The rBuLIF was purified to homogeneity from the total cell lysate of COS-1 cells using a two-step affinity chromatography. The purified LIF was confirmed by western blot and mass spectrometer (MS/MS). Particularly, high-resolution MS has identified the rBuLIF with 73% of sequence coverage with highest confidence parameters and with the search engine score of 4580. We determined the molecular weight of rBuLIF protein to be 58.99 kDa and 48.9 kDa with and without glycosylation, respectively. Moreover, the purified rBuLIF was verified to be functionally active by measuring the growth inhibition of M1 myeloid leukemia cells, revealing a maximum inhibition at 72 hours and half-maximal effective concentration (EC50) of 0.0555 ng/ml, corresponding to a specific activity of >1.6×10^7^ units/mg. Next, we evaluated the effect of rBuLIF on buffalo mammary epithelial cell lines for its role in involution and also identified the IC50 value for BuMEC migrating cells to be 77.8 ng/ml. Additionally, the treatment of MECs (BuMEC and EpH4) displayed significant (*P* < 0.05) reduction in growth progression, as confirmed by qRT-PCR analysis, suggesting its strong involvement in the involution of the mammary gland *in vivo*. Thus, we conclude that the glycosylated rBuLIF, purified from COS-1 cells was found to be functionally active as its natural counterpart.

## Introduction

Growth factors (GFs) and cytokines are the essential molecules for the proper execution of the cell proliferation and differentiation. Leukemia inhibitory factor (LIF), a member of the interleukin-6 (IL-6) family is a pleiotropic glycoprotein and multifunctional cytokine that acts on a wide range of cell types including osteoblasts, hepatocytes, adipocytes, neurons, embryonic stem (ES) cells, and megakaryocytes [1]. It was initially recognized to induce the differentiation of myeloid leukemia M1 cells into macrophages [2, 3] and later was reclaimed to function as an inhibitor of differentiation of ES cells [4]. However, based on the cell type and stage, it plays multifaceted roles such as regulation of growth, differentiation, inflammation, proliferation, and apoptosis. One such crucial function is its involvement in the maintenance of general cell growth progression and lineage-specific differentiation.

Various tissue-specific biological roles of LIF were reported such as involvement in the survival of neurons [5, 6], stimulation of osteoblasts [7], and regulation of hematopoiesis [8]. Based on such biological importance, recombinant human LIF (rhLIF) was also used for the clinical trials in advanced cancer patients to avoid chemotherapy-induced peripheral neuropathy, or implantation of the embryo into the uterus in infertile women [9, 10]. Embryonic stem cells (ESCs) have the capability to differentiates into a wide range of cell types and provide the paradigm for a mammalian stem cell that undergoes balanced self-renewal in culture [5]. Even though after the continuous efforts of several research groups, no buffalo ES cell line was generated and maintained for multiple passages [11–16]. One of the reasons for limited success in Buffalo and other large animal is due to non-availability of available buffalo LIF and knowledge of signalling pathways information, especially about species-specific LIF to ESCs pluripotent markers and culture conditions [16]. All these available information provide the powerful foundation to have the sources of highly purified and biologically active rBuLIF, in large quantities. Buffalo ESCs have plentiful application in the field of animal sciences and biotechnology related to targeted gene manipulation, transgenic studies, biomedical and agricultural research [17]. Unfortunately, previously none of the experiments was successful to establish the buffalo embryonic cell line from the inner cell masses (ICM) of blastocysts because it cannot be passaged for a long time *in vitro*, and these cells were called as ES-like cells; probably due to the use of a mouse or human LIF [16]. It is envisaged, but not limited that *in vitro* cultivation and production of bovine origin LIF provides the opportunity for culturing and maintenance of buffalo ESCs and it might improve in near future with this purified rBuLIF.

The possible reason for a limited understanding of buffalo LIF is also might be due to scanty information is available, that too only at the nucleotide sequence level in NCBI (Accession numbers: JN088208, NM_001290925). In silico analysis of LIF reveals that it is a highly glycosylated protein with six potential N-linked and six O-linked glycosylation sites (S1 and S2 Figs respectively). Also, at present, the commercially available recombinant LIF is produced in a bacterial host (*E*. *coli*). To overcome the limitation of bacterial host expression system where protein refolding and post-translation modifications are compromised, we developed a stably transfected COS-1 cell line that overexpressed glycosylated rBuLIF. Keeping in view the requirement of LIF in agricultural and animal sciences, its primary role for cell growth progression and cell lineage-specific differentiation and also to understand the various signalling pathways behind its pleiotropy, rBuLIF protein was purified from COS-1 cell line and its functional implication in mammary gland involution was studied *in vitro* in Buffalo Mammary Epithelial cell line (BuMEC) [18]. Its expression and purification were confirmed by qRT-PCR, western blot and mass spectrometer analysis. The biological activity of purified rBuLIF was checked on M1 myeloid cell line for its differentiation using BrdU assay. The real-time PCR analysis showed LIF regulates the expression of the transcription factor of proliferation markers of cell cycle regulators and in turn induces globule-shaped structure formation for unknown function (believed to be involved in involution) in mammary epithelial cells of mice and buffalo. To the best of our knowledge, this is the first report available about the purified rBuLIF to homogeneity purification and its potential application in *in vitro* expression system.

## Materials and methods

### Culture of stably transfected COS-1_BuLIF cell line and scale up

M1 myeloid leukaemia cell line (Cat. Code ATCC-TIB-192) and mouse mammary epithelial cell line (EpH4) (Cat. Code ATCC-CRL-3063) was purchased from American Type Culture Collection (ATCC, Virginia, U.S), Buffalo Mammary Epithelial Cell line was developed in our lab (BuMEC cell line) [18], and COS-1 cell line was procured from NCCS Pune, India. For transfection the COS-1 cells were cultured in growth medium containing DMEM supplemented with 10% FBS, 2mmol/L L-Glu, and antibiotics (Penicillin 100 U/mL, Streptomycin 30 μg/mL). They were incubated at 37°C in humidified atmosphere containing 5% CO2. The monolayer became confluent 4–5 days after seeding 1x10^6^ cells/flasks (25cm^2^ flasks), and the cells were sub-cultured at a split *ratio* of 1:3 by trypsinization (0.5% trypsin and 0.05% EDTA). The medium was changed every alternate day. We previously reported the in-depth protocol for the construction of recombinant pAcGFP1-N1 LIF vector and its stable expression in COS-1 cells [19]. Briefly, the transfection was performed using the PolyFect transfection reagent (Qiagen, cat. No. 301105). Initially, the 5x 10^4^ cells were seeded in an individual well of 6 wells plate. The recombinant rpAcGFP_BuLIF plasmid of 600 ng was added in 25 μl serum-free medium. Separately, 3 μl transfection reagent was taken in 50 μl OptiMEM medium and incubated for 5 min at room temperature followed by mixing together and again incubated at room temperature for 20 min. The prepared 75 μl complex was added to the cells and incubated for 12 h at 37°C in 5% CO_2_. After the completion of the incubation, the medium was replaced with fresh DMEM+10% FBS and permitted to grow for subsequent 36 h. Followed by the selection of transfected cells using G418 (400μg/ml) antibiotic. The cells were continuously grown in the presence of antibiotics until only resistant colonies were survived.

### Purification of rBuLIF from COS-1 cells

For purification of soluble rBuLIF, the transfected COS-1_cells harbouring pAcGFP1-N1_BuLIF expression constructs were cultured in T1000 (EMD, Millipore) for 5 days to attain the ~85% confluency. Cells were trypsinized and the harvested suspension was washed two times with ice-cold PBS by centrifuging at 4000 g for 15 min. The obtained cell pellet was initially lysed in PBS buffer containing 1% Triton X-100 and 1x complete mammalian mini protease inhibitor mix (Sigma) using mild vortexing. The ruptured cells were incubated on ice for 15 min for the extraction of soluble proteins and centrifuged at 20,000 g for 20 min at 4°C. The supernatant was discarded and obtained pellet was dissolved in 50 mM sodium phosphate buffer, pH 10, containing 0.5% SDS. To improve the pellet solubilization, the mixture was ultrasonicated with conditions—32 amplitude, 5 s pulse, on/off for 10 min. Again insoluble debris was pelleted by centrifugation for 10 min at 20,000 g at 4°C. After centrifugation NaHCO_3_ was added to the supernatant to a final concentration of 500 mM and pH was adjusted to 8.3 and the cleared lysates were used for purification.

The supernatant of recombinant protein cell lysate was loaded into CnBr-activated Sepharose 4B beads (GE-Pharmacia) coupled with the monoclonal antibody GFP following manufacturer’s protocol with few modifications. Briefly, prior to GFP mAb binding the Cn-Br Sepharose 4B beads were activated freshly. The 5000 mg CnBr beads were swelled in 15 mL coupling buffer (0.1M NaHCO_3,_ 0.5 M NaCl, pH 8.3) for 15 min (5000 mg swells about 15 mL final volume). The final volume was washed with 150 mL ice-cold binding buffer and spun at 1000 g. The supernatant was removed and 25 mL of cold binding buffer was added, along with GFP mAb and then rotated the mixture end-over-end for overnight at 4°C. After spinning, excess mAb from the supernatant was removed and the remaining active groups were blocked by 15 mL ice-cold blocking buffer (0.1 M Tris-HCl, pH8.0) at 4°C for 30 min. Final Sepharose/GFP mAb mix was rinsed with 15 mL ice-cold 1x PBS and the activated beads were packed in the column and connected to Akta Prime purification system (GE Healthcare, USA) and equilibrated. The 10 mL of pre-cleared BuLIF transfected COS-1 cell lysate supernatant was added with protease inhibitors cocktail onto GFP mAb-coupled affinity column. The column was washed with 10 column volumes with binding buffer PBS, 0.13 M NaCl, 0.01 M Na_2_HPO_4_, 0.01 M NaH_2_PO_4_, pH 7.4). The GFP-tagged BuLIF protein was gradient eluted (0–60%) using binding plus elution buffer (0.1M Glycin-HCl pH 4.5). The eluted fractions were immediately neutralized by adding, 1 M Tris-HCl pH 9.0 and fractions were dialyzed in ice-cold, 0.15 M PBS at 4°C for 1–2 h. The protein fractions collected were subjected to SDS-PAGE.

### Deglycosylation and western blot analysis for determination and validation of molecular weight

Affinity purified rBuLIF was confirmed by western blot using an antibody against human LIF (Merk Millipore, MAB4306). To determine the exact molecular weight of buffalo LIF protein with and without glycosylation the purified LIF protein was treated with PNGase F enzyme (NEB, P0704S). The PNGase enzyme was dissolved in sterile nuclease-free water to prepare 1mU/μl, which was used at a final concentration of 1 mU per 10μg of rBuLIF. The mixture was incubated at 37°C overnight in a closed microcentrifuge tube. The reaction was stopped by adding 1 M HCl for acidifying the mixture to pH 2. Resultant deglycosylated and glycosylated purified rBuLIF proteins were used for western blot analysis as per the procedure described in [19].

### Mass spectrometric analysis

The mass spectrometric analysis was performed according to standard lab protocols by the National Dairy Research Institute Mass Spectrometry facility [20, 21]. In brief, protein samples were subjected to SDS–PAGE and gel band was cut and washed once with Milli-Q water followed by three times with 500 μl solution containing 40 mM ammonium bicarbonate and 40% acetonitrile in a ratio of (1:1) for 15 to 30 min on a mixer. The gel bands were dehydrated by the addition of 500 μl of 100% acetonitrile. Disulfide bonds were cleaved by incubating the samples for 60 min at 60°C with 200 ul of 5 mM DTT in 40 mM ammonium bicarbonate buffer. Alkylation of cysteines was performed by the addition of 200 μl of 20 mM iodoacetamide in 40 mM ammonium bicarbonate buffer and incubation for 10 min at room temperature in darkness. Gel bands were covered with trypsin solution (12.5 ng/μl in 50 mM ammonium bicarbonate buffer). Proteolysis was performed overnight at 37°C and stopped by adding 5% formic acid. The prepared peptides were reconstituted and analyzed by Q-TOF Bruker Mass Spectrometer (Maxis HD). Acquired experimental data were subjected to ProteinScape with Mascot, Maxquant with Andromeda and Trans-Proteomics Pipeline (TPP) with Comet search engine to search the primary sequence database.

### Cell proliferation assay in the presence of purified rBuLIF protein

Standard M1 myeloid leukaemia cell line (ATCC-TIB-192) was used to determine the biological activity of purified rBuLIF. These cells were treated with 20 ng/ml of rBuLIF in parallel to normal cells (untreated) in a triplicate and BrdU cell proliferation assay was performed according to manufacturer’s protocol using assay kit (Cat No. Q11A58, Calbiochem, USA). Briefly, cells were seeded in a 96-well plate as 100 μl medium containing the density of 1×10^5^ cells/ml per well. The effect of rBuLIF on cell proliferation was assessed at every 12-hour interval up to 72 hours. The incorporation of BrdU was carried out after treatment by incubating the growing cells with BrdU reagent for 2 hours followed by stopping the reaction and measuring the absorbance at 540 nm using Nano-quant plate reader (Model: Infinite M200 PRO, TECAN). For the assay, cells were seeded in three biological replicates with three technical replicates resulting in nine measurements (n = 9) for individual labelling experiment. All the samples were seeded and processed in a similar manner during time-scale measurement of cell proliferation with BrdU assay. The data were presented as mean ± SEM. A cell proliferation graph was prepared by plotting the absorbance of rBuLIF treated and untreated cells against the respective hours of culture.

### Biological activity of the purified rBuLIF protein

The growth inhibitory effect of purified rBuLIF protein was measured on M1 myeloid leukaemia cells by its ability to induce the differentiation [2, 3]. M1 cells were routinely grown in RPMI 1640 medium with 5% fetal bovine serum (FBS). For comparison, the rBuLIF purified and commercial rhLIF, (Cat. No. LIF1010, Merk Millipore) were incubated with 1×10^4^ cells seeded in a 96-well microplate in 100 μl medium at a final concentration ranging from 0.0001 to 20 ng/mL in discrete increment of 10 folds. Relative inhibition in proliferation was calculated at the end of 72 hours of seeding at all the concentrations by BrdU assay as previously described. The M1 cell growth in terms of percentage was calculated by [(Absorbance 540 rBuLIF-Absorbance 540 without rBuLIF)/Absorbance 540 without rBuLIF] ×100. Finally, the effective concentration (EC50) of rBuLIF was determined. The data represent mean ± SD of three independent experiments.

### Cell migration assay

Effect of recombinant LIF was analyzed for the migration of buffalo mammary epithelial cells (BuMEC cell line) using the trans-well migration assay. Briefly, migration chamber was inserted in 24-well plate and 100 μl of serum-free media DMEM was added in the chamber followed by the addition of the 2.5×10^5^ /ml cells in 200 μl of serum-free medium. The complete medium 750 μl of DMEM containing serum was added in lower chambers with varying concentration of recombinant LIF from 0.001 to 100 ng/mL was used and cells were incubated for migration at 37°C for 24 hours in a humidified CO_2_ incubator. After incubation, the chambers were disintegrated, and cells were fixed using 3.7% formaldehyde for 2 minutes at room temperature. Then formaldehyde solution was removed by two times washing with PBS. The cells were permeabilized using 100% methanol at room temperature for 20 minutes followed by two times washing with PBS. Lastly, cells were stained with Giemsa at room temperature in dark for 15 minutes. The extra stain was removed by washing with PBS two times and then the non-migrated cells from the top side of the wells were removed by wiping the membrane with a cotton swab. The cells were counted using an inverted compound microscope and the number of migrated cells was presented as a mean value of three separate measurements ± standard deviation.

### qRT-PCR assays for proliferative and apoptotic genes in mammary epithelial cell lines (BuMEC) in the presence and absence of rBuLIF

Two cell lines namely, buffalo mammary epithelial cell line (BuMEC) and mouse mammary epithelial cell line (EpH4) were treated with purified rBuLIF and commercial human LIF proteins (40ng/ml) (Cat. No. LIF 1010) and incubated for 72 hours for comparison of the morphological changes. The BuMEC was previously established cell line in our lab and EpH4 was purchased from ATCC (ATCC-CRL-3063) both these cell lines were cultured using DMEM (Sigma) supplemented with 10% FBS (Hyclone, Logan, UT, USA), 200mM l-glutamine (Invitrogen). Subsequently, RNA was isolated from the cells; cDNA was prepared and analyzed for qRT-PCR for the proliferation and apoptosis marker gene expression. Briefly, specific primers were designed from selected proteins (S1 Table) GAPDH and β-actin were used as reference gene for internal control. The reactions method was set 5 min denaturation at 94°C followed by 35 cycles (at 94°C for 30 sec, at 61°C for 30 sec and at 72°C for 30 sec) with the completion of reaction at 72°C for 10 min. This cycle was followed by a melting curve analysis, baseline and cycle threshold values (Ct values) were automatically determined. Simultaneously, negative controls including mock reverse transcriptase without RNA or reaction mixture with nuclease-free water were checked for amplification and amplified products were separated on 1.8% agarose gel. Identification of fold change was calculated using 2^–ΔΔ^CT method. The t-test statistic was used for comparison of the expression of the gene at significance level ≤ 0.05). Data were analyzed using MS Excel 2007 and Prism software 5.01 (GraphPad Software, USA).

## Results

### Expression and purification of rBuLIF from COS-1 cells by affinity chromatography

**Fig 1:**
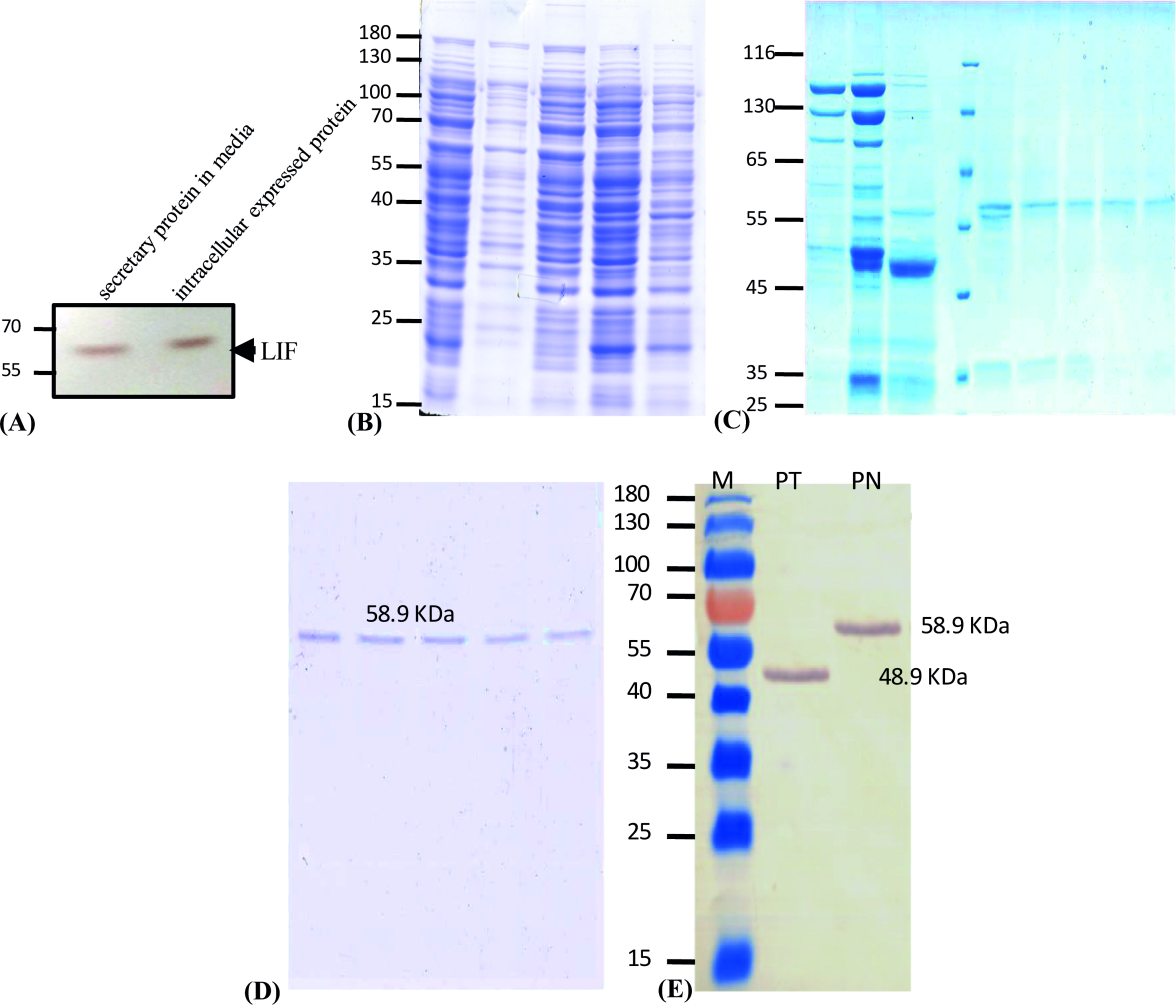
Expression of LIF in COS-1 cells. **(A)** GFP-tagged full-length LIF was expressed in COS-1 cells and detected by western blot as a secretory protein in the media as well as in total cell lysate. (B) SDS-PAGE Profile: Gel showing the initial washing fractions of the rBuLIF protein during affinity purification of whole cell lysate from the first run of purification. (C) Initially, collected fractions during the chromatographic run from Lane 1, 2 and 3 all the proteins obtained were concentrated in a lyophilizer 1:20 folds to visualize clearly in the SDS gel. M lane for the marker, first run elution profile of the peak shown in the Fig 2A-lanes 4–8. (D) The second run of the chromatographic elution: Eluted fractions from the first run were subjected to the same packed column for the separation of the proteins, based on the affinity. (E) Western blot image of purified rBuLIF protein for confirmation. M lane for the marker, PT lane for PNGase F treated BuLIF without glycan moieties showing 10 kDa reduction in size, PN lane for non-PNGase treated showing intact purified rBuLIF protein of 58.9 kDa molecular weight.

High quality total RNA was isolated from cumulus oophorus cells of buffalo oocyte and a full-length amplicon of 609bp was cloned into a pJET1.2 vector and transferred into expression vector pAcGFP-N1 (S3 Fig). Stably transfected COS-1_pAc_BuLIF_GFP was developed by retrograde passaging and a pure line of rBuLIF_GFP expressing COS-1 cell was used for purification of rBuLIF_GFP. The detailed description of the method has previously been reported [19]. rBuLIF was detected in concentrated media (100 times) as well as in cell lysate (20 μg total protein) by western blot against GFP protein (Fig 1A). The presence of secretory signal peptide in the rLIF protein resulted, its secretion in the medium; however, the obtained expression was very low. This allowed us to use the total cell lysate for purification using the following protocol (in detail description was provided in the material and method section).

**Fig 2:**
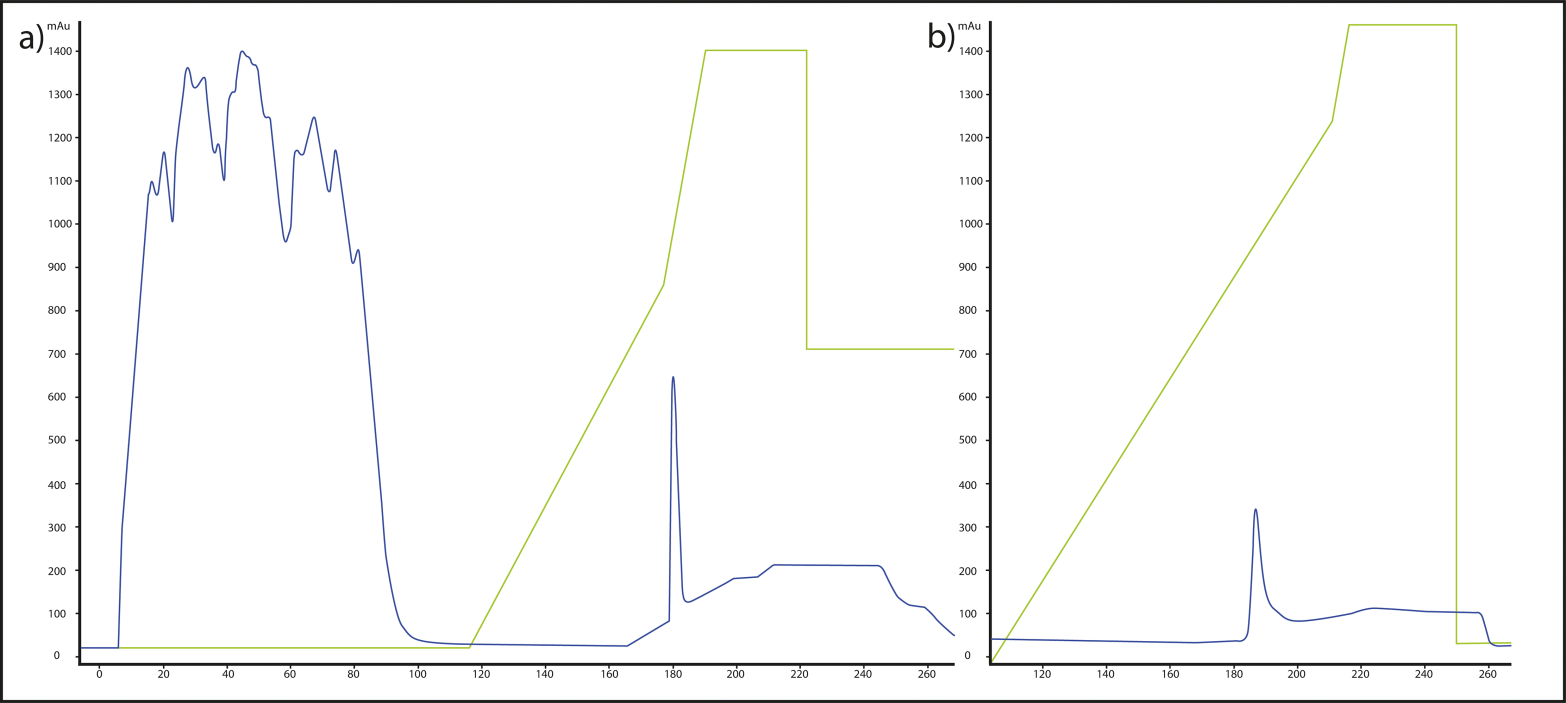
Chromatograms of rBuLIF-GFP protein during affinity purification using Anti-GFP antibodies from whole cell lysate. (A) First run of chromatography for purification (SDS profile shown in Fig 1B and 1C). (B) The second run of the purification, sample obtained from the first run peak was injected into the column for the second round of purification (respective SDS profile is shown in Fig 1D). The blue line in the chromatogram indicates the elution profile of the proteins while the green line shows the gradient applied.

The rBuLIF produced in the mammalian cell was successfully purified to homogeneity by the sequential chromatographic method. Initially, to simplify the procedure the mild lysis was carried out in PBS buffer containing 1% Triton X-100 (PBS lysis buffer). During this step recombinant LIF was found insoluble (data not shown). After high-speed centrifugation (20,000 g for 10 min) rBuLIF was identified in the pellet. The obtained supernatant part of soluble proteins was carefully removed which helped us to separate out many of the contaminating proteins prior to purification. The resultant pellet was dissolved in 0.5% SDS containing 50 mM sodium phosphate, pH 10. Finally, the prepared lysate was used for LIF purification using CnBr-activated Sepharose 4B beads bound with the monoclonal antibody against GFP. The recombinant protein was bound to the column via the antigen-antibody interaction, and the contaminating proteins were washed off from the column with 50 mL of binding buffer containing PBS, 0.13 M NaCl, 0.01 M Na_2_HPO_4_, 0.01 M NaH_2_PO_4_, pH 7 as gel showing the initial washing fraction of the total cell lysate during affinity (Fig 1B). The chromatogram showed broad fused peaks during washing, followed by a steep increase in gradient elution protein (0–60%) (Fig 2A). SDS–PAGE analysis of the peaks showed that the affinity bound proteins were gradient eluted during elution. After the first step of chromatography, the purity of the eluted protein was greater than 85%, as determined by Coomassie staining of SDS–PAGE gels (Fig 1C).

All the fractions were pooled and the remaining impurities were removed by loading into the same recharged affinity column (Fig 2B). Further second step chromatography resulted in a single peak of the pure protein evaluated by SDS–PAGE gel. The purity and identity of the LIF protein were lastly evaluated by Coomassie-stained SDS–PAGE and by Western blotting (Fig 1D and 1E). With this procedure, we achieved up to 95% purity for the recombinant buffalo LIF protein, without degradation products and with an overall yield of 0.4 mg per 1.45×10^8^ COS-1 cells.

### Deglycosylation and western blot analysis for determination and validation of molecular weight

Bioinformatics analysis of LIF protein sequence using two available online tools; GlycoMine (http://www.structbioinfor.org/Lab/GlycoMine) and NetNGlyc 1.0 server (http://www.cbs.dtu.dk/services/NetNGlyc) depicted the identification of six potential glycosylation sites on 31, 56, 85, 95, 118 and 138^th^ amino acid residues (S1 Fig) which would have increased the mol wt. of the LIF protein. To determine the exact molecular weight, we have deglycosylated the purified LIF protein by a PNGase enzyme which removes all the glycan moieties from the protein. Glycosylated and deglycosylated LIF protein was simultaneously used for the western blot analyses using the anti-LIF human antibody as primary Ab and a secondary antibody conjugated with horseradish peroxidase with Di-amino benzene (DAB) system as substrate. The presence of single bands in the blot confirmed the molecular weight with 48.9 kDa deglycosylated and 58.9 kDa glycosylated rBuLIF and also provides the validation that the purified protein was rBuLIF and no other contaminating protein was present in the preparation (Fig 1E). This finding also reports that presence of glycan moiety adds up additional 10 kDa mass to the LIF protein.

### Liquid chromatography-mass spectroscopy (LC-MS) confirmation of the purified protein rBuIFN-T

Recombinant purified LIF was in-gel digested and generated peptides were subjected to mass spectrometer analysis to confirm the identity of the protein. The database was searched for LC-MS generated precursor ions against three search engines (Mascot, Comet and Andromeda) provided the amino acid sequence coverage of about 73% with very high mascot score ≥4580. This pointed out that purified rBuLIF protein was highly pure and identity of protein was confirmed. However, the 22 amino acid initial signal peptide was not detected (Table 1).

**Table 1:** Mass spectrometer based database match of tryptic peptides. The database was searched using PSM, protein, site decoy fraction FDR values of 0.01 minimum peptide length 7 amino acids. Confidences of the identification of peptides were shown by lowest PEP, score, Intensity, and high MS/MS counts.

Length	Missed cleavages	Mass	Position	Charges	PEP	Score	Intensity	MS/MS Count	Sequence
08	1	1018.592	100–107	2	1.24E-38	66.692	2969700	4	ARIVEIYR
22	1	2487.302	34–55	5	0.013539	84.105	22227	5	EVMVAMYKIFAFINASIGNITR
09	0	1030.559	146–154	2	1.68E-133	96.027	804250	9	GIISNVICR
12	0	1465.692	38–49	2	8.62E-230	111.86	7447300	17	HPCPSNIMNQIR
26	2	2966.423	155–180	4	3.04E-211	109.23	2414200	4	ICSKYHVSHVDVTYGPDTSGKDVFQK
08	0	887.4899	183–190	2	1.95E-28	92.659	1033200	4	IGCQIIGK
14	0	1460.835	108–121	2	1.98E-47	71.879	151340	5	IIAYIGASIGNITR
17	1	1832.016	108–124	3	2.48E-19	59.728	1898800	4	IIAYIGASIGNITRDQK
18	1	2058.108	71–88	4	0.010172	220.826	17332.6	8	INATIDIMRGIISNVICR
09	0	1001.551	137–145	2	1.24E-220	104.01	2336700	23	INTTADVIR
18	1	2014.099	137–154	3	1.29E-05	45.221	101150	6	INTTADVIRGIISNVICR
12	0	1334.71	125–136	2	1.75E-230	114.02	16957000	5	VINPYAHGIHSK
17	0	1860.864	159–175	4	0.000346	60.724	4392900	4	YHVSHVDVTYGPDTSGK
22	1	2478.182	159–180	4	0.000297	62.936	108190	6	YHVSHVDVTYGPDTSGKDVFQK
12	1	1349.771	191–202	2	0.000106	45.829	80391	4	YKQVIAVIAQAF

### Determination of biological activity of rBuLIF using cell proliferation assay

**Fig 3:**
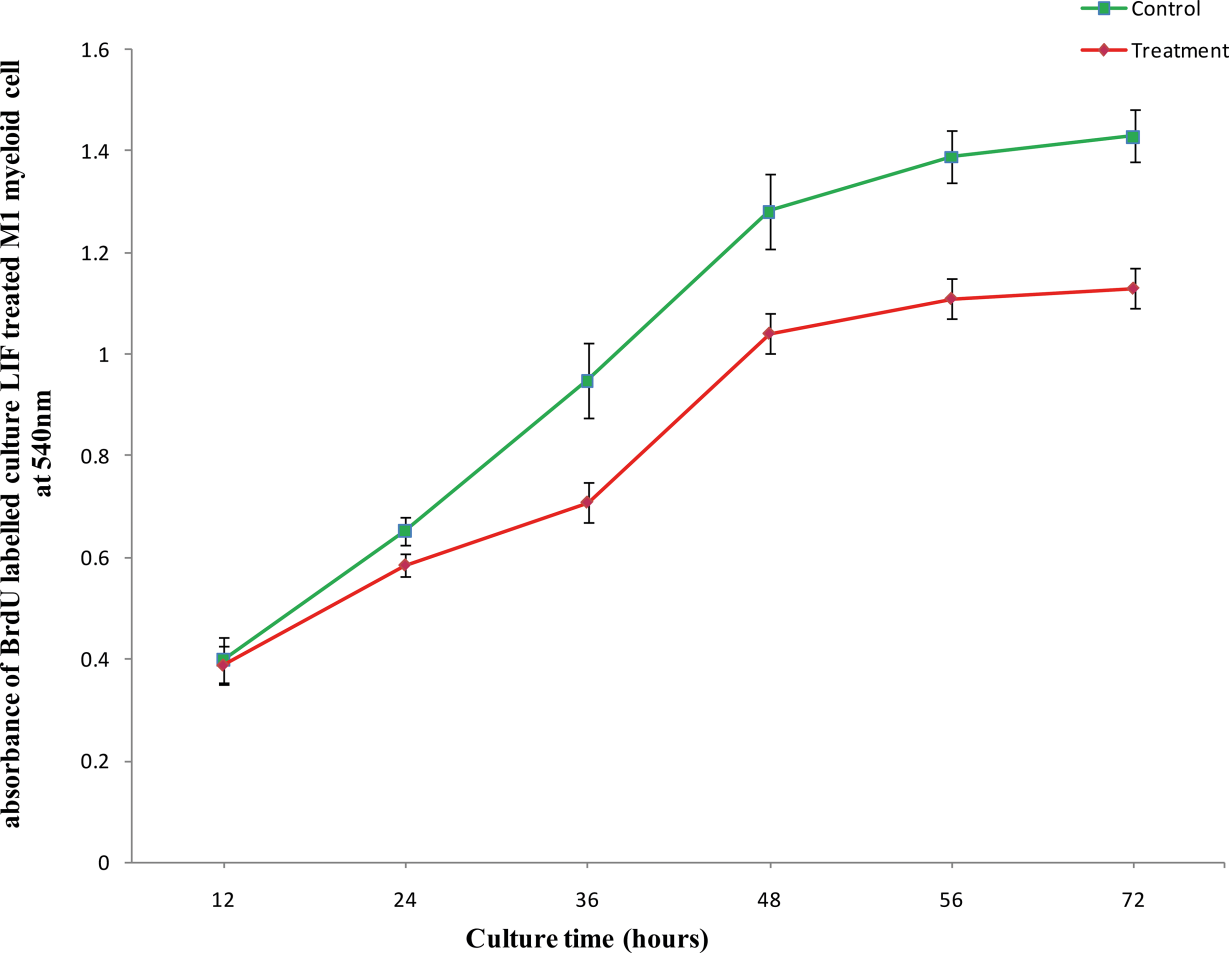
Cell proliferation assay of active purified rBuLIF on the timescale. The inhibition of growth of murine myeloid leukaemia M1 cells was measured by BrdU assay on different time points. The error bars indicate the standard deviation.

To demonstrate the biological activity of purified rBuLIF protein two different assays were performed. In the first assay, the M1 myeloid leukemic cells were used and treated with 20 ng/ml of LIF for 12 to 72 hours for differentiation into macrophages. The cells were seeded with 1×10^5^ cells/ml per well density and proliferation was analyzed at every 12 hours of intervals by BrdU cell proliferation assay based on the incorporation of 5-bromo-2'-deoxyuridine (BrdU). The assay results suggested that M1 cells treated with LIF had a minor reduction in the growth after 24 hours of the culture. Nevertheless, a significant change in the proliferation rate of M1 cells was seen from 36 to 72 hours of culture medium. The normalized curves suggested lowest growth rate difference at 72 hours of culture, after reaching maximal growth with LIF treatment in comparison to highest growth was observed on 72 hours without LIF treatment. The decrease in growth rate of M1 cells in culture, with LIF treatment, was steeper after 36 to 48 hours followed by nearly stationary growth up to 72 hours (Fig 3). Overall, the data indicate that rBuLIF impeded the growth of M1 cells had *vis-a-vis* control cells (No BuLIF) which evidently showed that purified LIF was biologically active.

**Fig 4:**
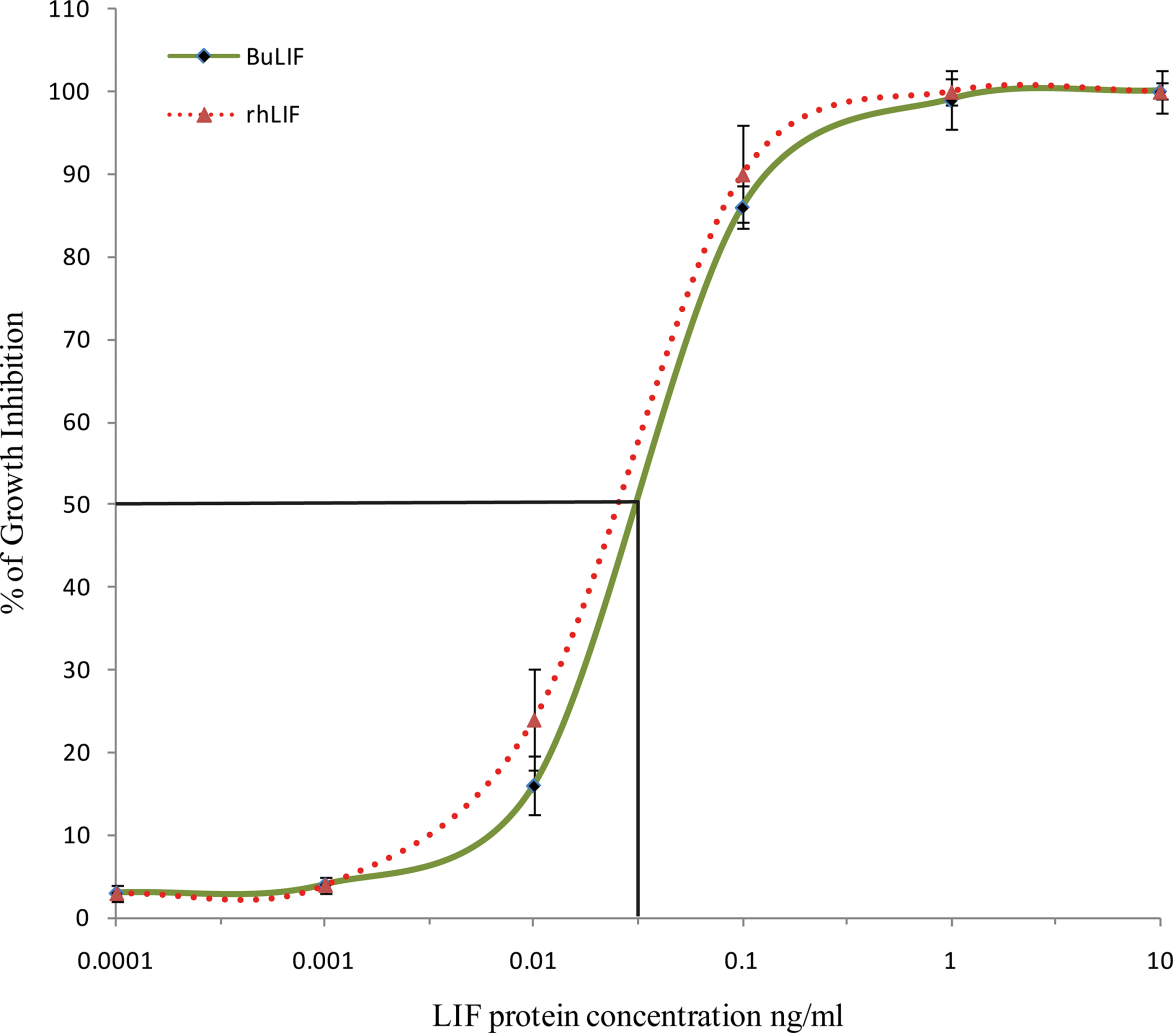
Bioactivity assay of rBuLIF on murine myeloid leukaemia M1 cells by BrdU assay. Comparison of growth inhibition of M1 cells incubated with purified rBuLIF (solid line) and commercial rhLIF (dotted line).

In the second assay, the biological activity of the purified mature rBuLIF was compared with commercially available rhLIF. For this, different concentrations of purified LIF protein were tested for the stimulation of M1 cells (1×10^5^ cells/ml per well) grown in 96 well plate. The activity of LIF can be characterized on the basis of its ability to cause differentiation of M1 myeloid cells [2, 3]. The results exhibited an apparent inhibition of cell growth in a dose-dependent manner, at a comparable level to the commercially available recombinant hLIF (Fig 4). The inhibitory percentage growth of M1 cells was proportionally decreased with increasing concentration of LIF protein. The EC50 value (50% effective concentration) of purified rBuLIF, based on this assay was determined to be 0.0555 ng/mL, corresponding to a specific activity of >1.6×10^7^ units/mg. This calculated specific activity of rBuLIF was found similar to other recombinant preparations [3, 22, 23].

### Analysis of the functional activity of recombinant LIF through Cell migration assay

**Fig 5:**
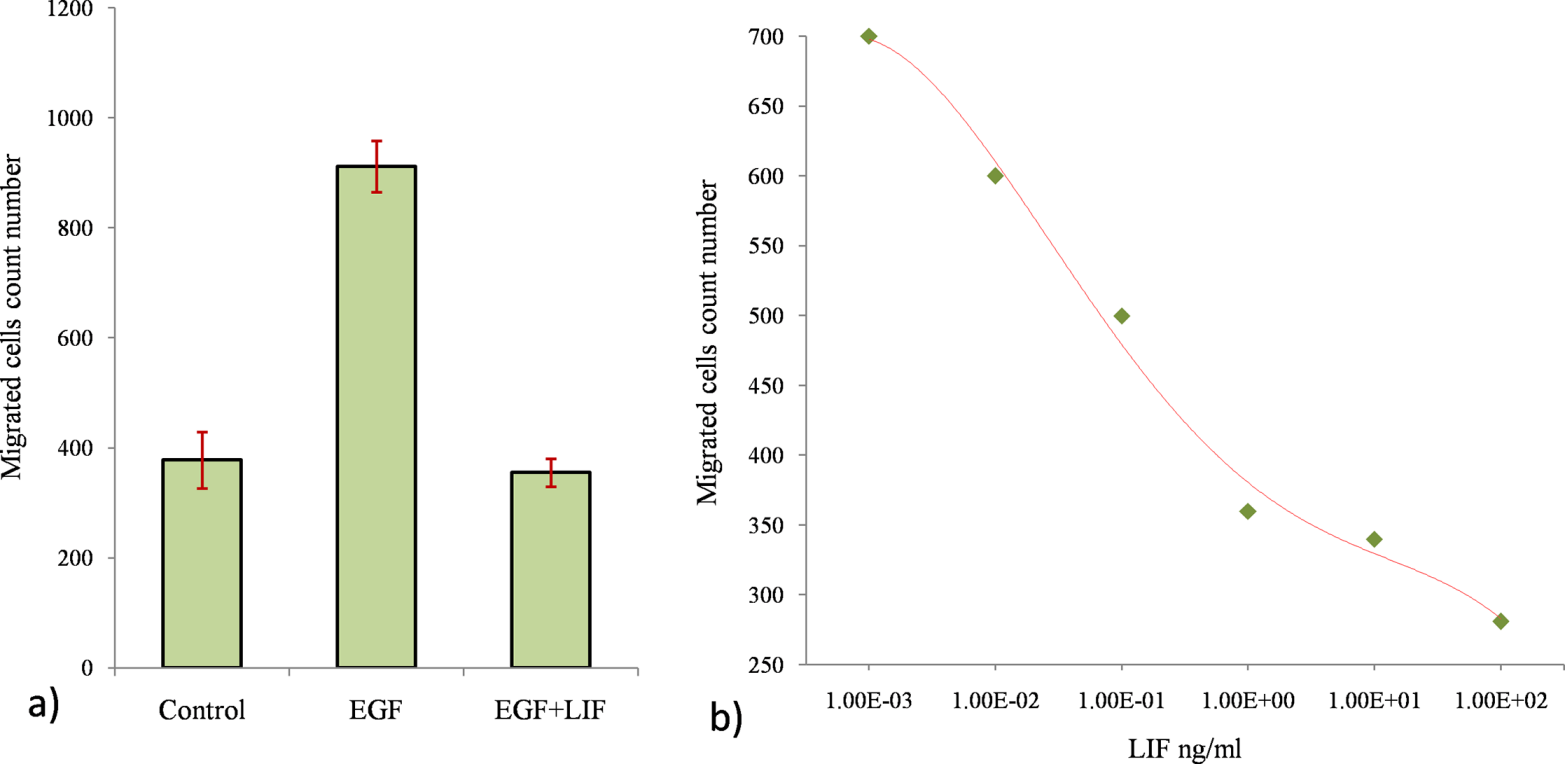
Cell migration inhibitory effect of rBuLIF on buffalo mammary epithelial cells. (A) Cells were induced by addition of EGF (50 ng/mL) as a stimulant for migrating cells in migration assay that increase the migration rate approx. 2.421-fold. In the presence of rBuLIF protein (100 ng/mL), the EGF induced migration of the BuMEC cells was completely abolished. (B) Determination of IC50 value for the cell migration inhibition effect of rBuLIF. Increasing concentrations of rBuLIF protein were used in the cell migration assay which was plotted against the number of migrated cells and the best fit inflexion point was determined for the IC50 value of rBuLIF.

Additionally, migration assay on buffalo mammary epithelial cells (BuMEC) was performed to analyze the functional biological effect of rBuLIF. The migration of BuMEC cells was induced by EGF alone and its combination with the rBuLIF, which was added to analyze the migrating cell numbers. The migration of BuMEC cells was stimulated with 50ng/mL EGF proteins in assays along with the absence or presence of rBuLIF at a concentration 100 ng/mL in a migration chamber. For control, BuMEC cells were incubated without EGF or rBuLIF proteins. In comparison to control, the EGF induced migration of BuMEC cells increases approx. 2.412- fold (912 cells ± 40 after EGF treatment in comparison to 378 cells ± 45 in the control). On the contrary, the addition of purified rBuLIF to BuMEC cells completely diminishes the EGF induced migration (385 cells ± 24) (Fig 5A).

Finally, we calculated the IC50 value of rBuLIF protein for the function of inhibition of cell migration. For this, the BuMEC cells were treated as above and the inhibitory effect of rBuLIF protein was analyzed at various concentrations plotted Fig 5B. The graph showed rBuLIF decade Log concentration against the number of migrated cells and with the help of sigmoidal fitting procedure an IC50 value of 77.8ng/ml for rBuLIF protein was determined (Fig 5B). These results gave clear evidence that the rBuLIF protein was functional as it inhibited the EGF induced cell migration.

### Analysis of morphological and biological changes in the presence of LIF on mammary epithelial cell lines

**Fig 6:**
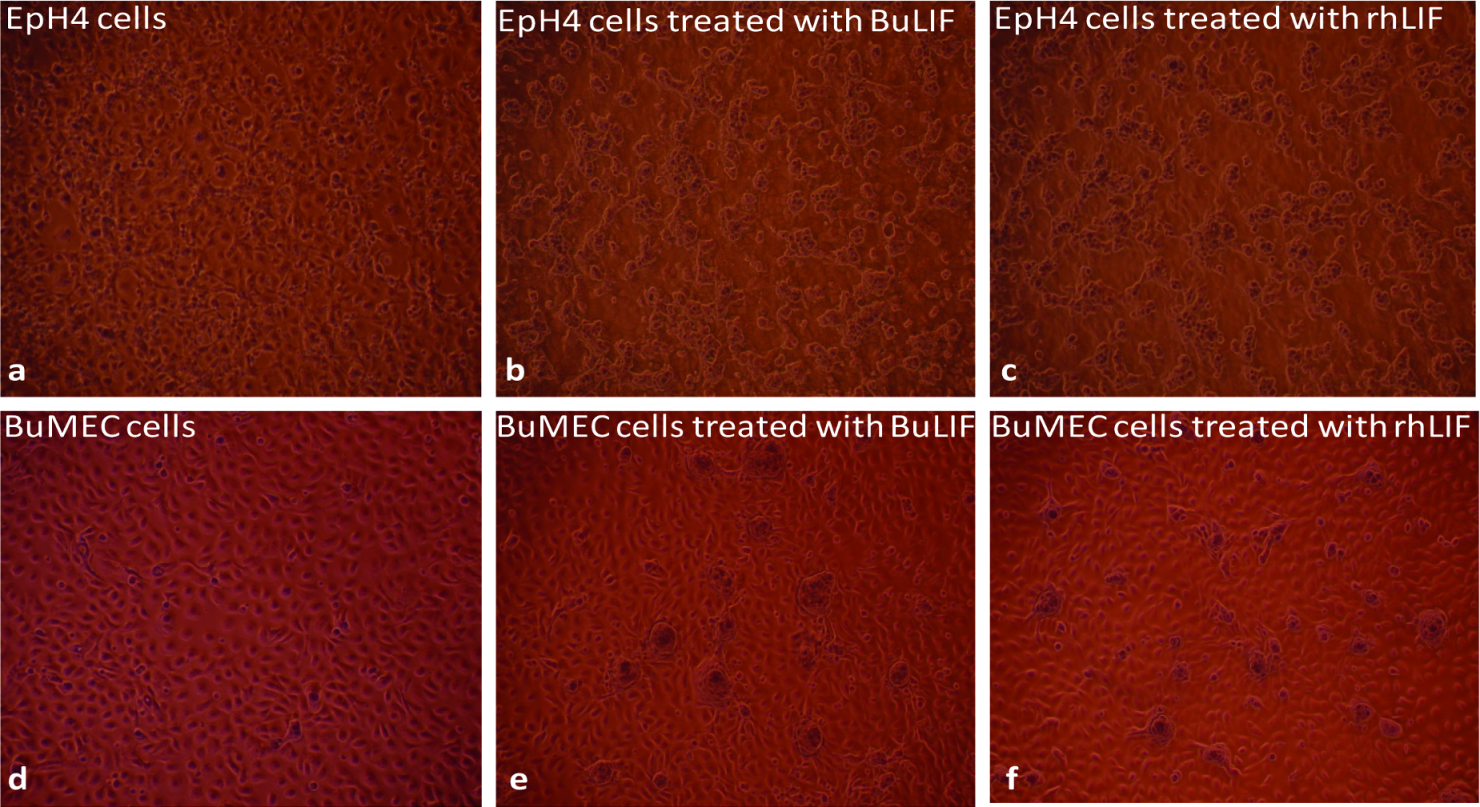
Treatment of rBuLIF to mammary epithelial cells. (A) Mice mammary epithelial cells–EpH4 without rBuLIF treatment as a control. (B) EpH4 cells treated with purified rBuLIF exhibit changes in morphology forming globule structures. (C) EpH4 cells were treated with commercially available rhLIF. (D) Normal buffalo mammary epithelial cells (BuMEC) in full confluency under bright light. (E) BuMEC cells treated with rBuLIF also exhibit changes in morphology forming globule structures. (F) BuMEC cells treated with rhLIF also showed changes in morphology used as a positive control under bright light. Yellow arrows indicate the globular structures. Scale bar, 500 μm and 10X magnification.

**Fig 7:**
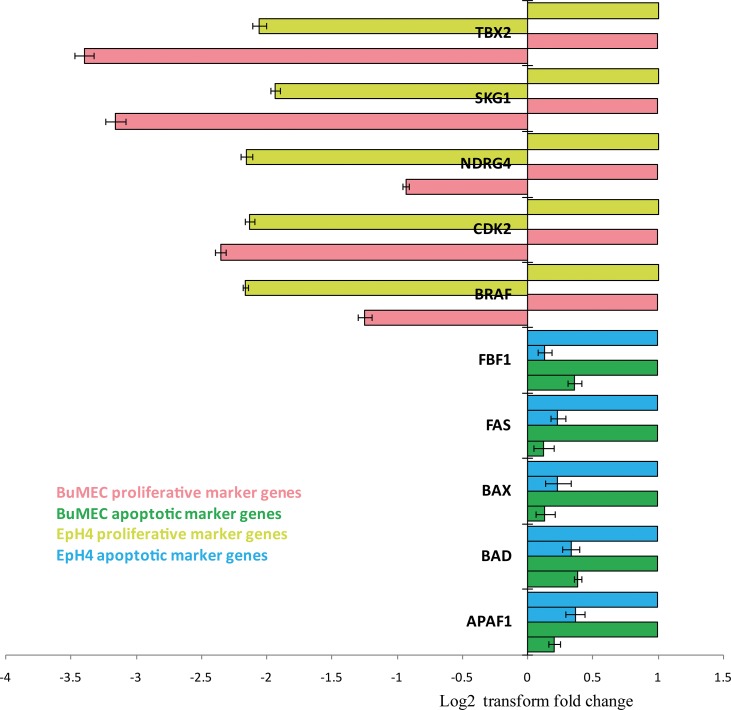
Real-time PCR analysis of proliferative and apoptotic marker genes in rBuLIF treated mammary epithelial cells. Proliferative and apoptotic marker genes were analyzed after treatment of rBuLIF on BuMEC and EpH4 cells. Fold changes were represented in the form of log2 transform values.

Considering the important role of LIF in involution process of mammary gland developmental process, we treated two different mammary epithelial spontaneously transformed cell lines, originally isolated from mice and buffalo (EpH4 and BuMEC respectively) with rBuLIF. Further, we compared the rBuLIF protein to commercially available rhLIF. After 72 hours of treatment, mammary epithelial cells both of the cell lines displayed a change in the morphology and different acinus/dome-like structure formations in the *in vitro* grown cells compared to control. These structures were seen in both types of LIF used in the treatment (Fig 6). To test whether rBuLIF mediated changes in cells structural physiology (dome-like structures), we analyzed the relative expression level of 5 proliferative and 5 apoptosis marker genes through quantitative PCR (Fig 7). These qRT-PCR results showed high down-regulation of proliferative genes (average all genes were found to be 0.2 fold change) while there was the slight decrease in the expression level of apoptosis maker genes in LIF-treated cells compared to control. This finding suggests that the LIF treatment for 72 hours on MEC cells slowed the cell growth rate by reducing the expression of proliferation genes (Fig 7). To understand and discover the LIF-mediated downstream effects, its regulatory mechanism to form globule-shaped structures, an in-depth study is required to be undertaken. Together all of these biological assays in this study have confirmed that purified rBuLIF protein retains its biological activities and is functionally equivalent to commercial rhLIF.

## Discussion

Leukemia inhibitory factor (LIF) is a pleiotropic glycoprotein, which performs various biological activities in a highly context-dependent manner. For instance, LIF induces the differentiation in M1 cells, whereas inhibits the differentiation of embryonic stem cells [24, 25]. It plays a vital role in embryonic implantation via activating selective pathways, such as PI3K/AKT and JAK/Stat3, depending on the cell type and situation [26–30]. Its expression in the mouse uterus at the time of blastocyst implantation is important. Females which do not have a functional LIF gene are although fertile; their blastocysts are incompetent for implantation [26]. Recombinant LIF is also under clinical trials as a driving force for the embryo implantation in the uterus of women who fail to conceive. During the menstrual cycle, its expression in the uterus is confined to the glandular epithelium during the mid and late secretory phases [31, 32]. At the time of mammary gland involution during tissue remodelling, it is a driving key for the progression of involution [33]. During this process, the spatiotemporal interaction between epithelial and stromal cells is the prerequisite. The crosstalk between these two cell types is mediated by various growth factors among which LIF has been implicated to play an important role in the regulation of the involution processes [33]. It is the physiological activator of STAT3 in mammary gland and it is observed that LIF plays a dual role. It activates STAT3 –mediated apoptosis during involution and ERK1/2 –mediated branching morphogenesis during pregnancy [34]. Despite all of the aforementioned physiological significance recorded in human or mouse developmental biology, its functional role in animal science is scanty and fewer investigations have been conducted. Recently, we detected the up-regulation of buffalo LIF in the early stage of pregnant cotyledonary cells (< 45 days) in comparison to caruncular cells which suggested its crucial value in the continuation of pregnancy [35].

One of the foremost focuses of this work was to purify the recombinant active, accurately processed, rBuLIF protein in its native state. The full-length rBuLIF protein consists of 202 amino acids, out of which 29 leucine and 6 cysteines are present which resulted in three disulphide bonds [36]. Moreover, we found that protein has a pI value of 9.44 which means it is highly positively charged molecule at physiological pH due to the presence of 20 basic amino acids (arginine and lysine) residues. Therefore, it can be presumed that the expression of this protein in the bacterial host system might result in misfolding-mediated aggregation, turning into insoluble inclusion bodies and loss of function [37]. Previously, human LIF expressed in the bacterial system resulted in a high concentration of inclusion bodies [23]. Refolding of inclusion proteins into native form is a time-consuming process and without assurance that the obtained protein will regain its native conformation with proper biological activity. Moreover, the important limitation of the bacterial host system is the unavailability of PTMs. rBuLIF possesses 6 potential sites for N-linked glycosylation modification (S1 Fig). Therefore, the mammalian expression system was chosen for heterologous expression of rBuLIF.

In this study, the rBuLIF protein was expressed with a 22 amino acid signal peptide. In spite of signal sequence, LIF secretion in the medium was very small in comparison to intracellular level. It was probably due to the difference in the pH of cytoplasm and culture growth medium. Since rBuLIF has a pI value of 9.44, this difference could prevent the protein from being secreted. However, increasing the pH of the media may not improve protein secretion but may lead to increased cell death. Therefore total cell lysate was used to purify LIF from the transfected COS-1 cells.

Initially, cell lysis was performed by two consecutive steps. Firstly, the COS-1_BuLIF cells were lysed using mild Triton X-100/PBS buffer, in which LIF was in pellet while the majority of contaminating cytosolic, as well as membrane proteins, was in the supernatant (data not shown). In the second step, the obtained pellet was solubilized in phosphate buffer at pH 10 by addition of SDS which resulted in the dissolution of LIF protein. Fascinatingly, phosphate buffer alone or with SDS without adjusting the pH to 10 were incapable to dissolve the rBuLIF after the first lysis step. This might be due to the consequence of the high net negative charge on the rBuLIF at this pH, and its plausible binding to genomic DNA. However, raising the pH to 10 along with a combination of SDS, the solution swiftly turned viscous. The viscosity of the solution was reduced by ultrasonication and then rBuLIF protein was straightforwardly purified to homogeneity by mAb-based affinity chromatography in two steps.

The identity of the purified rBuLIF protein was confirmed by a mass spectrometer with 73% of sequence coverage. But in the MS data, we did not identify the 22 amino acid signal peptide sequence. This results may be due to either the signal peptide was completely cleaved at some stage in the cellular sorting of proteins or due to the low intensity of signal peptide precursor ions in MS.

We further examined that the purified rBuLIF was biologically active causing the differentiation of M1 cells in the direction of macrophage lineage. We also showed that rBuLIF inhibits the migration of the buffalo mammary epithelial cells and totally abolished the activity of the EGF for migration in comparison to control. Finally, considering the imperative role of LIF in involution process of mammary gland remodelling, we analyzed this purified protein for their effect on MECs and concluded through qRT-PCR that it slows down the cell growth. Our results consistently indicated that purified rBuLIF demonstrates properties equivalent to those reported for the native protein. We confirmed the functional tests using M1 myeloid cells and mammary epithelial cells. In addition, the rBuLIF protein produced in this study can be useful in numerous applications in veterinary science like in improving implantation, bovine stem cells and developmental biology. The accessibility of rBuLIF will undoubtedly foster novel research findings to investigate the mechanism of LIF action e.g. the characterization of orphan LIF-receptor, and the biological miscellany properties of LIF, such as its decisive involvement for proliferation, migration or differentiation of different cell types. This will certainly assist to untangle the complex biological role of this molecule in vivo. This report also opened the door for a new era of animal stem cell and regenerative medicine research. Taken as a whole, our strategy offers not merely an efficient and quick technique to generate biologically active rBuLIF but also might be pertinent to produce additional different growth factors and cytokines.

## Supporting information

S1 Fig Prediction of in silico N-linked glycosylation residue and consensus peptide sequence. N-glycosylation is known to Asparagines residue which occurs in the Asn-Xaa-Ser/Thr stretch (where Xaa is any amino acid except Proline). While this consensus tripeptide (also called the N-glycosylation *sequon* in many texts) may be a requirement, it is not always sufficient for the Asparagine to be glycosylated. Furthermore, there are a few known instances of N-glycosylation occurring within Asn-Xaa-Cys (a Cysteine opposed to a Serine/Threonine at the N+2 position). The NetNGlyc 1.0 Server—DTU CBS was used with the URL *www*.*cbs*.*dtu*.*dk/services/NetNGlyc/*
(TIF)

S2 Fig Prediction of in silico O-linked glycosylation residue. The amino acid residues in the list are potential glycosylation sites, showing their positions in the sequence and the prediction confidence scores. The sites with scores ≥**0.5** are predicted as O-Linked glycosylated and marked with the string "#POSITIVE" in the comment field. The NetOGlyc 4.0 Server—DTU CBS was used with the URL *www*.*cbs*.*dtu*.*dk/services/NetOGlyc*
(TIF)

S3 Fig RNA isolation and confirmation through PCR. a) Agarose gel electrophoresis of total RNA isolated from cumulus oophorus cells of buffalo oocyte shows the 18S and 23S rRNA bands b) PCR amplification product of BuLIF of 609bp size c) PCR analysis for confirmation of LIF insert in ampicillin resistant *E*. *coli* colonies, 1 kb DNA ladder (Lane M) and PCR products of LIF amplified from the plasmids isolated from ampicillin resistant *E*. *coli* colonies 609 bp long BuLIF.
(TIF)

S4 Fig Full length amino acid sequence of BuLIF. The full length amino acid sequence of the protein BuLIF and the prediction of the pI/Mw using online web tool expasy (https://web.expasy.org/compute_pi/).
(TIF)

S1 Table List of primer pairs employed in the study. Detailed information for the sequence of the primer pairs along with primer length.
(XLSX)
