# Supplementary figures and images for: Recombinant purified buffalo leukemia inhibitory factor plays an inhibitory role in cell growth
Source: PLoS One. 2018 Jun 13;13(6):e0198523. doi: 10.1371/journal.pone.0198523 (PMC5999108; doi:10.1371/journal.pone.0198523)

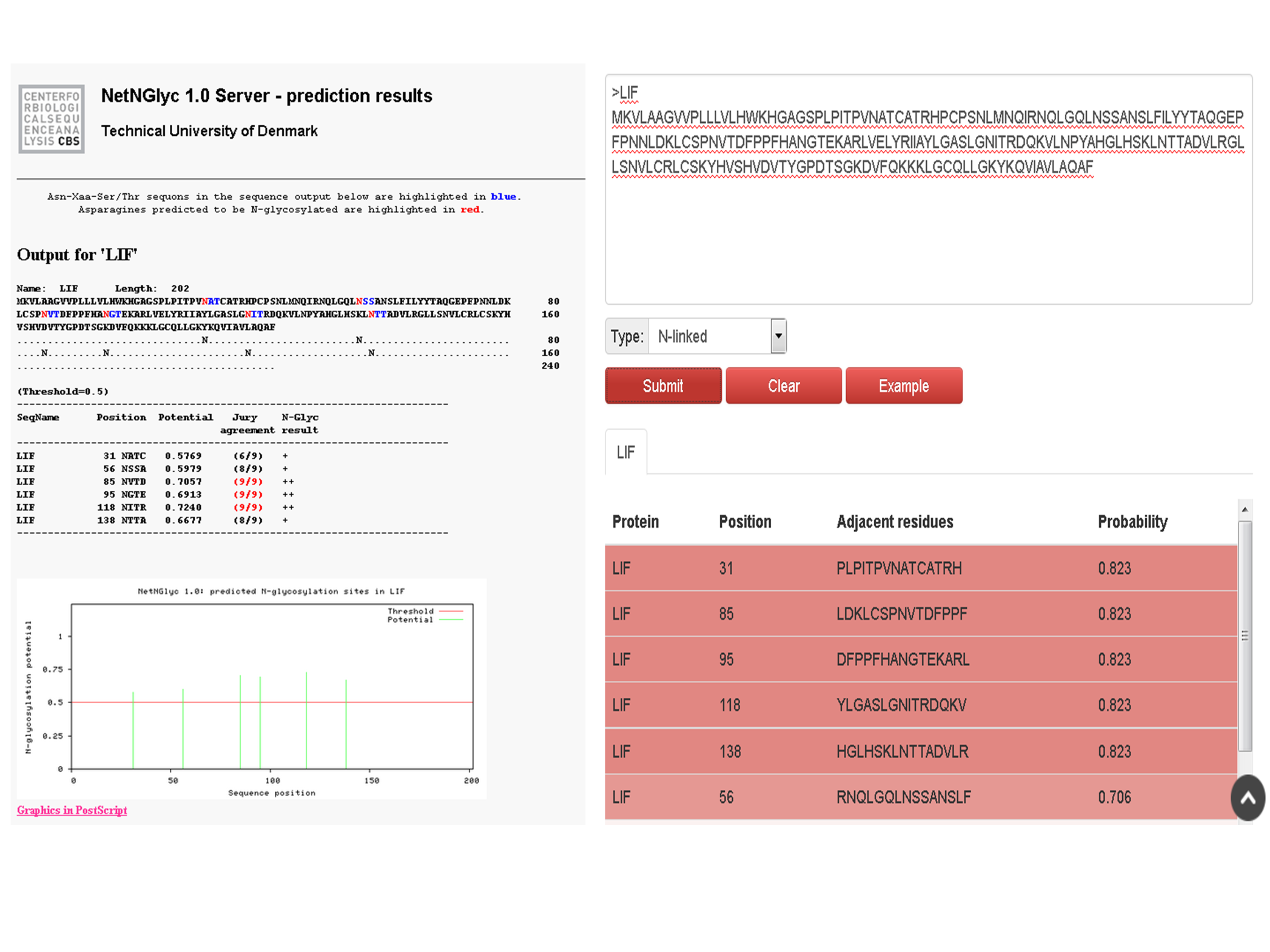

Supplement: S1 Fig — N-glycosylation is known to Asparagines residue which occurs in the Asn-Xaa-Ser/Thr stretch (where Xaa is any amino acid except Proline). While this consensus tripeptide (also called the N-glycosylation sequon in many texts) may be a requirement, it is not always sufficient for the Asparagine to be glycosylated. Furthermore, there are a few known instances of N-glycosylation occurring within Asn-Xaa-Cys (a Cysteine opposed to a Serine/Threonine at the N+2 position). The NetNGlyc 1.0 Server—DTU CBS was used with the URL www.cbs.dtu.dk/services/NetNGlyc/ (TIF) [file pone.0198523.s001.tif]

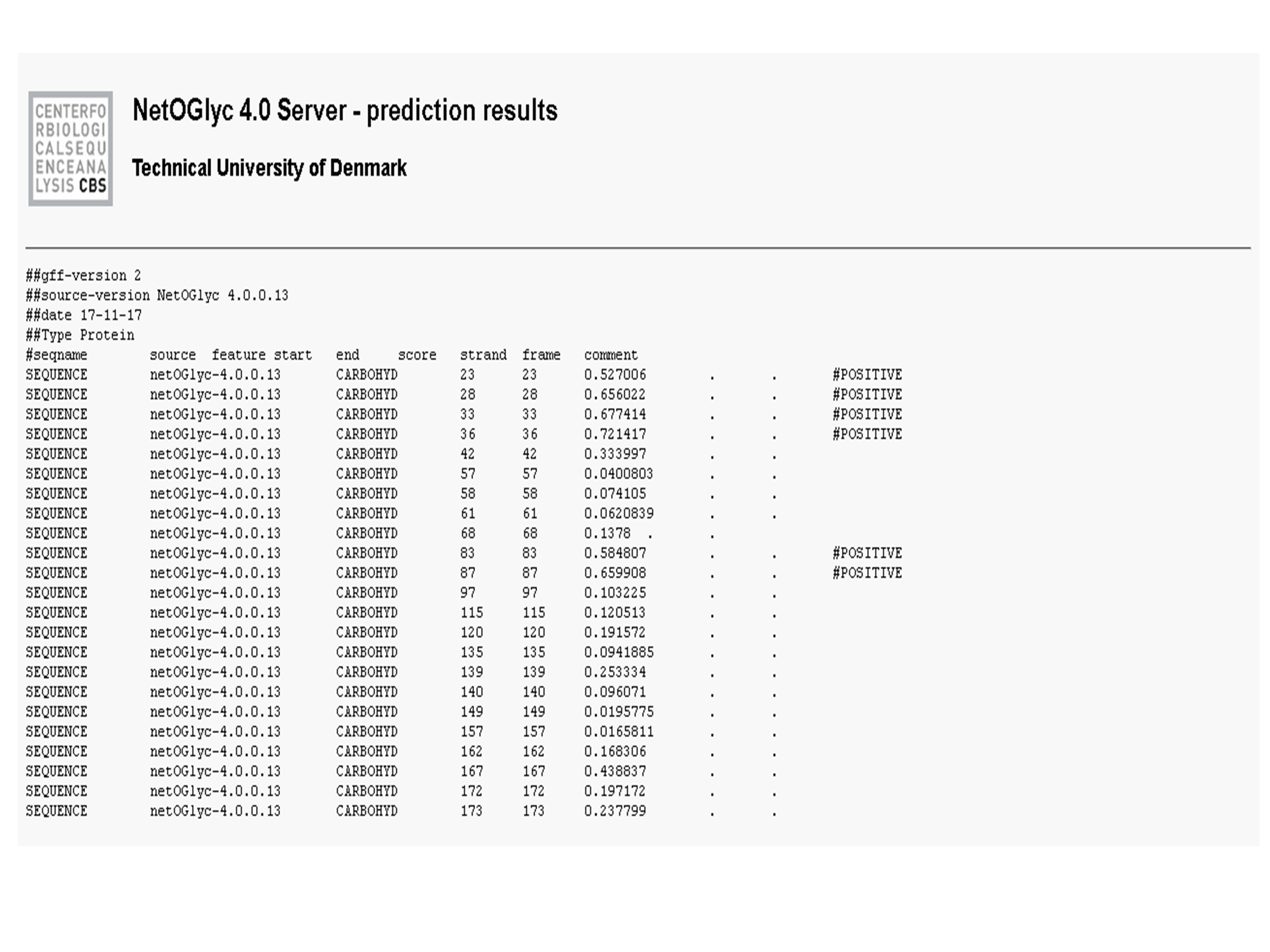

Supplement: S2 Fig — The amino acid residues in the list are potential glycosylation sites, showing their positions in the sequence and the prediction confidence scores. The sites with scores ≥0.5 are predicted as O-Linked glycosylated and marked with the string "#POSITIVE" in the comment field. The NetOGlyc 4.0 Server—DTU CBS was used with the URL www.cbs.dtu.dk/services/NetOGlyc (TIF) [file pone.0198523.s002.tif]

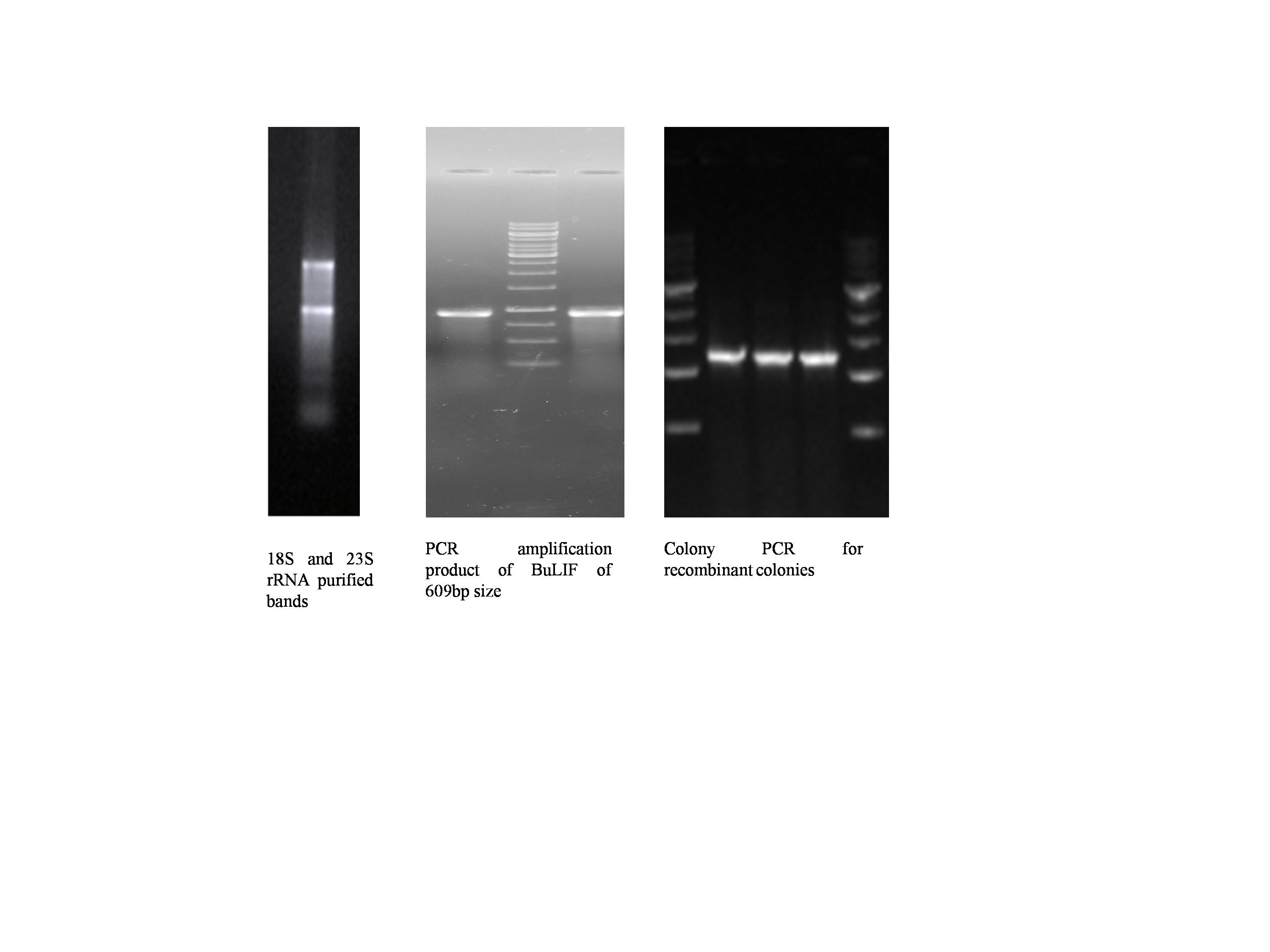

Supplement: S3 Fig — a) Agarose gel electrophoresis of total RNA isolated from cumulus oophorus cells of buffalo oocyte shows the 18S and 23S rRNA bands b) PCR amplification product of BuLIF of 609bp size c) PCR analysis for confirmation of LIF insert in ampicillin resistant E. coli colonies, 1 kb DNA ladder (Lane M) and PCR products of LIF amplified from the plasmids isolated from ampicillin resistant E. coli colonies 609 bp long BuLIF. (TIF) [file pone.0198523.s003.tif]

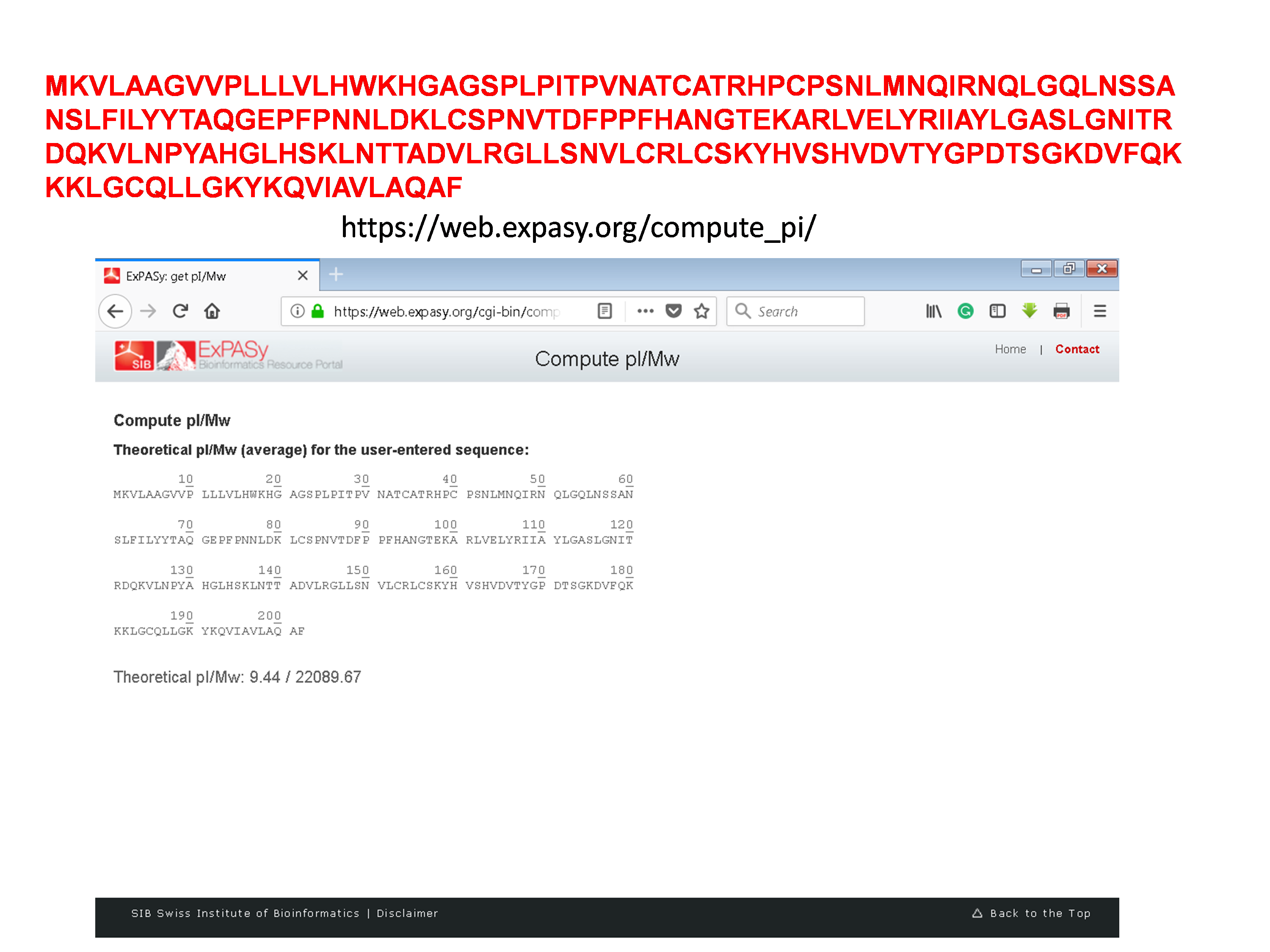

Supplement: S4 Fig — The full length amino acid sequence of the protein BuLIF and the prediction of the pI/Mw using online web tool expasy (https://web.expasy.org/compute_pi/). (TIF) [file pone.0198523.s004.tif]
